# An Evidence-Based Guide for Delivering Mental Healthcare Services in Farming Communities: A Qualitative Study of Providers’ Perspectives

**DOI:** 10.3390/ijerph21060791

**Published:** 2024-06-17

**Authors:** Rebecca Purc-Stephenson, Nicole Roy, Adachukwu Chimaobi, Deanna Hood

**Affiliations:** Department of Social Science, University of Alberta, Camrose, AB T4V 1R3, Canadachimaobi@ualberta.ca (A.C.);

**Keywords:** rural, mental health, agriculture, farmers, service delivery, qualitative

## Abstract

Individuals living in rural areas often face challenges in accessing healthcare, increasing their risk of poor health outcomes. Farmers, a sub-population in rural areas, are particularly vulnerable to mental health issues and suicide, yet they exhibit low rates of help-seeking behavior. The aim of our study was to develop an in-depth understanding of the issues influencing mental help-seeking among farmers living in rural areas from the perspectives of healthcare providers, as well as to explore the strategies providers use to navigate through these issues to effectively engage with this vulnerable population. Methods: We used a descriptive phenomenological approach to understand healthcare providers’ perspectives, experiences, and approaches to providing mental healthcare to farmer clients in rural areas. Semi-structured interviews were conducted with 21 participants practicing in Canada between March and May 2023. Results: Our analysis yielded five thematic areas: (1) ensuring accessibility, (2) establishing relatability, (3) addressing stoicism and stigma, (4) navigating dual roles, and (5) understanding community trauma. Conclusions: Healthcare service delivery for farmers is multifaceted. This study fills a gap in knowledge by translating these data to inform an evidence-based model and a list of recommendations for implementing agriculturally informed practices in rural areas.

## 1. Introduction

Compared to urban populations, consistent evidence shows a health disadvantage associated with living in rural areas, a trend observed across developed countries [1,2]. In Canada, research consistently reports higher mortality rates, decreased life expectancy, greater incidence and prevalence of morbidity, and poorer self-reported health status in rural populations compared to their urban counterparts [3,4,5,6,7]. Several explanations for this difference have been proposed, including a higher likelihood of smoking, alcohol use, and obesity [4,8], patterns of health service use [9,10], and socioeconomic factors such as lower levels of education, income instability, and exposure to hazardous environmental and occupational conditions [11,12]. Within rural areas exists one sub-population, farmers, who report concerning levels of poor physical and mental health. Given the difficulty of engaging with farmers on mental health issues [13], we aimed to explore the issues influencing mental help-seeking among farmers from the perspectives of mental healthcare providers. We were also interested in understanding the strategies these providers used to navigate these issues to effectively engage with this vulnerable population.

In 2021, nearly one-fifth of Canadians (18%) lived in a rural area [14]. The term “rural” is complex and socially constructed, with no universal definition [15]. For our study, we applied a broad definition that considers rural areas as all territories beyond urban areas [16]. Thus, rural areas can include towns, villages, agricultural spaces, as well as undeveloped and wilderness areas. Although rural areas share some similarities (i.e., small populations, located away from urban areas), they vary greatly in population densities and living conditions [17]. Moreover, there is growing recognition that rural areas contain sub-populations with distinct health risk factors, highlighting the need for targeted interventions [18]. One particular rural sub-population at risk for poor physical and mental health is farmers. Agriculture is recognized as one of the most stressful occupations [19]. Not only must farmers endure daily stressors, such as long work hours, manual labor, and time pressure, but they also face factors beyond their control such as fluctuating market prices, environmental regulations, and extreme weather conditions [19,20,21]. Farm stress has been linked to depression, anxiety, and burnout [22,23,24,25], as well as suicide [26]. A national study of over 1000 Canadian farmers revealed that 57.8% were classified with mild to severe depression, 49.2% with mild to severe anxiety, and they had elevated levels of burnout and low levels of resilience compared to the general population rates [25].

While timely help-seeking can mitigate the adverse effects of poor mental health [26], farmers have shown a lower propensity to seek treatment for physical or mental health issues compared to the general adult population [27,28]. In fact, for many farmers, seeking help often becomes a last resort, if pursued at all [26,29]. Apart from the relatively fewer healthcare providers offering mental health services in rural areas compared to urban locales in Canada [24,30], farmers exhibit reluctance to seek assistance due to various factors, including financial and geographical barriers, uncertainty about where to seek support or assess relevance, concerns regarding anonymity, and the enduring stigma surrounding mental health within farming communities [28,31,32,33,34]. A critical consideration for farmers when seeking mental health support is whether the services and providers possess “farm credibility”, indicating an understanding of the unique needs and challenges associated with agricultural life [35]. Such credibility may significantly influence the establishment of a therapeutic alliance, thereby impacting treatment engagement and outcomes.

Given the challenges many Canadians in rural and remote communities face in accessing mental healthcare services, coupled with the pressing issue of poor mental health among farmers, the Canadian healthcare system is actively seeking solutions to ensure equitable access to timely, relevant, and high-quality care [35]. While existing research on farmers and mental health services has primarily focused on their barriers to care [31,32,33,34,36], there has been comparatively little exploration of the perspectives of healthcare providers in rural areas and their strategies for engaging with farmers to address their mental health needs. One study conducted by Vayro et al. [29] explored how rural clinicians approached mental health issues with their farmer patients. The findings revealed that clinicians found it helpful to begin consultations by asking patients if they had other concerns, as this allowed farmers an opportunity to discuss topics such as family or farming stressors. Despite clinicians being a familiar point of contact for farmers [31], medical doctors typically have limited training in mental health to adequately support the complex mental health needs of farmers [32,37,38].

With a growing interest in expanding healthcare services to rural areas to address the mental health needs of farmers [35], it is imperative that these services effectively address farmers’ barriers to help-seeking and establish credibility within the agricultural industry. A recent environmental scan of mental health support services in Canada identified several successful initiatives engaging individuals in rural areas, particularly those from farming communities [30]. However, no study has yet explored help-seeking issues and successful strategies used for farming populations from the perspectives of mental healthcare providers. Therefore, employing a strength-based approach that includes the positive attributes rather than the negative ones [39], our study aimed to develop an in-depth understanding of the factors influencing mental help-seeking among farmers in rural areas from the viewpoint of mental healthcare providers. Additionally, we sought to explore the strategies used by providers to navigate these challenges and effectively engage with this vulnerable population. Our goal was to use this information to derive a set of lessons learned to inform interventions and practices of other healthcare providers interested in developing programs or services for individuals living in farming communities.

## 2. Methods

### 2.1. Design

We employed a descriptive phenomenological research design [40] to gain a deep understanding of healthcare providers’ perspectives and experiences in delivering mental health support to individuals in farming communities. Phenomenology was chosen as an appropriate approach because it enables researchers to learn about a topic in-depth from the direct, lived experiences of others [40] and explore how various contexts and backgrounds influence providers’ understanding. Because the goal of descriptive phenomenology is to describe the participants’ experiences and perspectives so that new insights are gained, researchers are required to set aside their preconceptions, and knowledge of theoretical frameworks, and empirical science during the analysis process [40]. This study was reviewed and approved by the University of Alberta’s human research ethics board (Pro00126443).

### 2.2. Participants

Drawing from a database that identified mental health support services for individuals residing in rural areas in Alberta, Canada [30], we randomly selected 25 services out of a possible 217 using a random choice generator. Each service provider received an email invitation from the first author to participate in a one-on-one semi-structured interview, aimed at exploring their perspectives and experiences in delivering mental health services to individuals in farming communities. Additionally, we advertised our study on social media platforms such as Facebook and our program website. Those interested in participating contacted the first author via email to schedule an interview. To be eligible to participate, individuals had to offer direct mental health support, serve a rural area, and have been operational for the past 12 months. We received 17 responses to the email invitations and 4 responses to our study advertisement. All participants were compensated with a $20 gift card from an online retailer.

### 2.3. Data Collection

Each interview began with a review of the study objectives and procedures, and participants provided verbal consent to continue. All interviews (N = 21) were conducted virtually via Zoom, audio-recorded, and lasted approximately 30 min. They followed a semi-structured protocol focusing on three main areas: (1) perspectives on the mental health needs of farmers, (2) approaches they used to address farmers’ barriers to mental healthcare, and (3) experiences providing mental health services in rural areas. The interviews were led by the first author, who had extensive experience conducting qualitative interviews. Field notes were taken during each interview and were added to each transcript as a memo to complement the qualitative analysis. Subsequent to the interviews, participants received a copy of the consent form and a mental health resource sheet tailored for the farming community via email. All interviews were transcribed verbatim.

### 2.4. Data Analysis

Prior to the analysis, all interview transcripts were screened for accuracy and any identifying information was removed to protect the identities of the participants. Transcripts were uploaded to Quirkos qualitative software to facilitate data analysis and data management. To ensure that our analysis was truly inductive and not informed by our assumptions and prior knowledge of the topic, our analysis team included two researchers (NR and AC) who had a limited understanding of the theory and research on the topic. Our group also met weekly to discuss the analysis. The interviews were independently reviewed and analyzed by three of the authors (RP, NR, and AC). Our analysis was guided by Colaizzi’s [41] seven-step process that included (1) reading and re-reading the transcripts to make sense of them; (2) identifying significant statements and phrases that relate to the topic; (3) describing the meaning from the statements and phrases; (4) moving statements and phrases into common themes and sub-themes; (5) compiling an exhaustive description of the themes and sub-themes; (6) refining and describing the themes so that the fundamental structure of phenomenon emerges; and (7) validating the findings through discussions with experts. We sought feedback from three experts, who included two registered counseling therapists and one general practitioner, all of whom lived and practiced in a rural area and had a background in farming (e.g., the therapists were from a farm family and the general practitioner lived on a farm). Further content validation for our findings came from achieving data saturation. Data saturation occurs when no further issues or insights are identified during data collection, and the data start to repeat, signaling that additional data collection is unnecessary and a sufficient sample size has been achieved [42]. In our study, data saturation began to occur at approximately participant number 15. The research team met weekly to review the coding process and addressed any discrepancies in coding through consensus.

## 3. Results

The analysis included interviews conducted with 21 participants who provided mental health services to farmers in rural areas. The sample predominantly comprised women (*n* = 19, 90.48%), with providers offering mental health support through various channels: community-based social service agencies (*n* = 10, 47.61%), private counseling practices (*n* = 9, 42.88%), or healthcare facilities (*n* = 2, 9.52%). Community-based support services and healthcare facilities provided mental health programs, support groups, workshops, and individual counseling, and all services were free for residents. Private counseling practices provided individual, family, and group counseling services that were paid for by the individual or through the patient’s insurance coverage.

Our sample included 7 mental-health program coordinators, 5 counseling therapists, 3 registered social workers, 2 mental-health program directors, 2 registered psychologists, 1 registered psychiatric nurse, and 1 nurse. On average, participants had worked in their current role for 8.24 years (*SD* = 6.84), with a range of 2 to 24 years. While 19 participants had lived in a rural area for their entire lives, the majority (*n* = 15, 71.43%) had a background in farming or agriculture. Specifically, 9 participants were part of a farm family, and 6 currently lived on a farm and identified as farmers.

Analysis of the interview data revealed five major themes that underscored the issues recognized by healthcare providers as needing attention to meet the needs of farmers. Each theme and sub-theme are described in detail below, accompanied by verbatim quotes from the participants.

### 3.1. Ensuring Accessibility

Participants emphasized the intertwining nature of geographical, technological, temporal, and financial barriers, creating a daunting landscape for farmers to seek help. In their view, many farmers struggled with the dilemma of prioritizing their own mental well-being amidst competing demands and limited resources (e.g., only owning one vehicle). Complicating matters was the tendency of many farmers to delay seeking care, so when they sought help, they tended to be in crisis. The need to provide accessible, timely services was highlighted by one participant, “For some mental health programs, it’s six months to a year wait to get in. So, I don’t know if you’ve ever had a mental breakdown. Can you wait six months?” (Participant 8).

Moreover, a lack of awareness or recognition of poor mental health symptoms often led farmers to only seek help when they reached a crisis point. For this theme, we identified three sub-themes: geographical proximity, scheduling conflicts, and financial considerations. For each, we highlighted how healthcare providers addressed the concerns.

#### 3.1.1. Geographical Proximity

The geographic isolation of smaller rural towns created a significant challenge for many farmers to access timely mental health services, underscored by the scarcity of specific services tailored to them. For instance, one healthcare provider explained, “Within a smaller community, right, you’re very far away from services, there’s just no services that are accessible. And the ones that are might not be the ones you need” (Participant 1). In particular, participants noted that mental health specialists and general practitioners were scarce in rural areas. One participant stated, “I don’t have a psychiatrist that I can refer people to if they need medication … I don’t have a family doctor that I can say is accepting new patients in the area … We haven’t had a new family doctor [in our area] in a decade” (Participant 6).

Given the distance to access services, many farmers needed to dedicate part of their day to traveling long distances sometimes to seek professional assistance. One healthcare provider observed, “I think travel is a big concern. I have some people that drive from [location] all the way to see me … that’s a solid commitment and traveling two and a half hours for family counseling” (Participant 21). Others noted that rural roads could be isolated and treacherous in winter, making travel that much more challenging and dangerous. Even though the winter months might be a slower season for many farmers, the long drive for services in potentially poor weather likely discourages many from seeking help. As one participant stated, “If they’re already stressed or struggling mentally, having to drive on the highway for over an hour in the winter when it’s −20 degrees temperatures is only going to add another layer to their stress. It’s risky, you know? I get why people feel stuck” (Participant 14).

#### 3.1.2. Ensuring Service Options

In response to accessibility challenges, many healthcare providers opted to continue offering virtual sessions post-pandemic. However, despite efforts to enhance accessibility, technological barriers such as poor internet connectivity persisted in rural and remote areas. In a digital era where online information is increasingly relied upon, healthcare providers acknowledged the unreliable nature of internet services, which frustrated them and their patients. As articulated by one healthcare provider, “When you get out more west from here, their internet service sucks. It doesn’t matter what provider you have, it’s terrible. They can’t connect with me and I can’t connect with them” (Participant 5). Moreover, some rural communities such as Hutterite colonies continued to have internet restriction rules, so healthcare providers would sometimes go to these communities or offer telephonic services.

While transitioning out of the pandemic, most service providers have tried to offer a broader range of in-person, telephone, and online options to improve flexibility. While healthcare providers acknowledged that telephonic or online services provided anonymity which was important in smaller rural areas, there was a common belief that farmers preferred in-person services. For example,

“It’s a mix. Of course, it was definitely more virtual when we first got started in 2020. But now, there’s definitely more people that are wanting to come to our physical office, which is good by us. So I would say it’s probably like an 80/20 split of 80% in-person, the other 20% virtual. We do have clients that will swap back and forth, depending on how their day is playing out or where they need to be and that kind of a thing” (Participant 6).

Participants agreed that the job structure of most healthcare providers, especially those working in healthcare organizations or community-based services, operated within standard 9 to 5 business hours, which do not align with farmers’ irregular work patterns. Participants stressed the importance of offering sessions outside of regular business hours and having flexible cancelation policies. As one participant highlighted,

“If [farmers] are available, it’s not necessarily during regular business hours … [They] might be stuck with seeding all day, or spraying at a very certain time because there’s a very small window for that. …Farming doesn’t operate on eight to five”. (Participant 1)

#### 3.1.3. Funding and Insurance Coverage

One of the most prominent issues impacting accessing programs was funding and insurance. From the healthcare providers’ perspective, the concern centered on the high cost of their services and the lack of insurance available for farmer clients to cover them. The limited coverage resulted in financial constraints for farmers, restricting their ability to access necessary counseling sessions or in some cases, limiting the number of sessions they could have for free. According to one participant,

“[P]sychologists know that they’re high in demand, so they’re $250 an hour, and then people only have $500 worth of benefits. So I charge $100 an hour, so I could give five sessions. They can only get two from a psychologist”. (Participant 8)

While some participants offered their services on a sliding scale, most emphasized the need for increased government funding to expand access to counseling services, advocating for subsidies or broader insurance coverage that includes non-psychologist services. Moreover, participants urged for a broader definition of mental health to enable community-based organizations to deliver programs beyond traditional counseling, such as recreational programs that incorporated mental health components. As one participant pointed out,

“More money. It always comes down to money. But I think the other thing is that it’s important to notice that there has been no increase in our funding since 2016. So populations have increased, the needs of people and community and all communities have astronomically increased, yet the amount of funding for preventative programs has remained the same”. (Participant 12)

Increasing funding and insurance coverage for mental health was viewed as a good way to communicate that mental health is just as important as physical health. Several participants noted that mental health was still seen as a weakness or personal flaw in farming communities, and having to pay out-of-pocket for counseling services reinforced the belief that they were dealing with unusual problems. One participant noted, “Funding is need to normalize mental health as much as possible, especially for the agriculture crowd” (Participant 18).

### 3.2. Being Relatable

Participants described the need for healthcare providers to find a way to relate to their patients, and often this meant translating their mental health service in a way that resonated with farmers and worked to establish credibility in the agricultural community. Our analysis revealed two sub-themes: building trust and connecting culturally.

#### 3.2.1. Building Trust

Participants emphasized how crucial it was to demonstrate an authentic understanding and appreciation of the struggles and stresses inherent in agriculture to establish a solid foundation of trust with farmer patients. For example, many participants explained how farmers often felt that their lifestyle and work were often misunderstood by healthcare providers unfamiliar with rural areas, often referring to them as “outsiders” or “city folk” who “don’t know the first thing about what we do”. As highlighted by one participant who stated,

“I think the relatability is so important. It’s part of the foundation to building trust. So, if someone says ‘I am working 14 h, and I feel like I’m running on my feet all the time, and I have no time to eat or anything’ and then the therapist says, ‘Well, why don’t you just hire somebody else?’ Like, if that’s not always possible depending on the operation or whether it’s a family farm”. (Participant 2)

Recognizing this, the healthcare providers we interviewed mentioned how important it was for their services to be tailored to the needs of farmers. They demonstrated that they could relate to farmers by having extended hours of operation, weekend sessions, and flexible cancelation policies. Many also mentioned that their website highlighted their background in farming or agriculture. In other words, healthcare providers needed to demonstrate their relatability in their business structure as well as their own knowledge. As one healthcare provider stated,

“There’s a lot of centralized programs that are, you know, a one-size-fits-all approach for delivering healthcare. I think farmers can see through that, and they will call bullshit on it. Basically, they’re thinking ‘this is not for me’, but you’re telling me it is. But when I pick up the phone and talk to you, you don’t know where I am or what I do” (Participant 5).

Participants highlighted a concerning trend of high turnover among counselors and healthcare providers in rural areas, which can significantly impact the trust-building process within farming communities. This turnover eroded the continuity of care and could undermine the efforts made by farmers who had invested their time, money, and effort in seeking help. The departure of counselors for urban opportunities created a revolving-door effect, leaving rural communities feeling abandoned and mistrustful of healthcare providers. Our participants mentioned that maintaining trust required an ongoing effort, and many gained it by being active members of the community. This point was emphasized by one participant,

“There are some communities that I served that had counselors come in and then take a position that opened up in the city. It’s a sort of revolving door, so then the community has a hard time trusting as well” (Participant 18).

#### 3.2.2. Connecting Culturally

Healthcare providers unanimously agreed on the importance of establishing cultural connections with farmers to foster trust and encourage service utilization. Several commented how people living in rural areas differed a lot from those living in urban areas, such that the “rural demographic wants a personal touch and wants to know that the person they’re talking to is human with their own life and their own problems” (Participant 13). Furthermore, a shared understanding of farming sometimes removes the psychological distance between the client and the provider, allowing them to address the issue directly. For example, one participant explained,

“Patients can talk about farming with me and I get it, right? They can tell me about, you know, seeding being a lot of pressure …. And I can say, ‘Yeah, I totally get that. I’m in an intergenerational farm, so I understand where you’re coming from [and] how that’s affecting your life’. Then we can move forward rather than focusing on giving me all the details” (Participant 14).

Relatability was a significant factor in establishing trust among farmers to use the services. For example, farmers were inclined to seek assistance from familiar faces within their community rather than outsiders, highlighting the importance of established relationships and community connections. For example, one participant explained,

“I think people’s [family] names really mean a lot too, so there’s that automatic trust built in, [because] the person who’s doing a program or service has a family name that you recognize”. (Participant 2)

### 3.3. Addressing Stoicism and Stigma

Healthcare providers acknowledged the concerns about privacy, stigma, cultural attitudes, and the perceived toughness within rural, farming communities. Many observed that farmers, especially men, were reluctant to seek help due to fears of judgment and gossip within close-knit communities. Our analysis revealed two sub-themes: acknowledging farm culture and managing mental health stigma.

#### 3.3.1. Acknowledging Farm Culture

Participants described how engaging and treating farmers was made easier by having an understanding of what farm culture entailed. In their words, farm culture has deeply rooted values of independence, stoicism, and toughness. Often, this means that work comes first and their needs second. Participants described how they would expect to have long gaps between patient visits given the seasonal work of farming. Others noted how they avoided scheduling workshops and events between April and October. While one of the strengths of farm culture is knowing others in your community, that level of familiarity sometimes represents a threat to privacy, discouraging farmers from seeking timely help. As one participant noted, “In a small rural community, everybody knows everybody and everybody knows your stuff. [Mental health] may be something you don’t want to talk about, or you might not want to be seen accessing a service like that because somebody’s going to see” (Participant 12).

Many participants observed that men in rural, farming communities grew up in a culture that valued and practiced self-reliance and determination and associated hard work with pride. One participant explained that reinforcing a flexible, confidential policy was important and provided this explanation about farm culture:

“You’ve got to tough it out. They’ll tell themselves [that] it’s not anxiety, it’s not depression, it’s farming. So you’ve got to suck it up and keep going. Don’t ask for help. If you ask for help, then there’s something wrong with you”. (Participant 7).

While these values were pervasive in farming communities, they were more prominent among men and often prevented many from seeking help even when they knew they needed it. As one participant explained,

“Men, you know, … they don’t want to go to the doctor for help. And especially the older folks, and when there’s large distances to cover and so much going on … a lot of these guys are suffering in silence” (Participant 4).

#### 3.3.2. Managing Mental Health Stigma

Some participants explained that their farmer patients often wanted to talk to someone that they could relate to, but they simultaneously did not want others to know they were seeking help. With the general agreement that stigma still existed in rural areas, one participant explained, “There’s way more fear of reaching out in the community. Because if I’m seen coming in these doors, I’m perceived as weak or less than, and I think that is amplified in the farming community” (Participant 7)

Most healthcare providers approached issues of mental health stigma by taking steps to “disguise” their mental health services if they did not offer online services. For example, one participant explained that if you wanted clients to come to your office, you certainly “wouldn’t have a big sign outside your door” that says “mental health”. She explained, “We have an office inside of the college. So people can park there and go anywhere in the college” (Participant 8). Others explained that because the need for anonymity was strong in farming communities, mental health programming was often embedded into more comfortable venues such as community dinners or wellness workshops.

While these strategies were generally successful at building awareness about mental health, they were not as powerful as word-of-mouth recommendations from farmers to their peers. Indeed, many healthcare providers recognized how powerful peer influence was in rural communities, and many relied on “having the word spread” about a good experience to decrease stigma and bring in new clients. For example, one participant explained:

“I think one of the first ranchers that I saw, you know, once he kind of put the pieces together like, ‘This is a really good resource’ and ‘Everybody should be doing this’. He would come in [and say] ‘I’ve been telling all my friends about this’. And then you start to see more of them filter through”. (Participant 6).

### 3.4. Navigating Dual Relationships

Our participants recognized that living in a rural area meant it was inevitable that they would encounter a client in the community. All healthcare providers stated that they discussed issues of confidentiality at the outset of their first session, with most telling their patients that if they saw each other in public, they would not initiate a greeting unless the patient did so first. However, in small rural communities, providers often found themselves in situations that tested their boundaries and forced them to maintain professionalism at all times. For example, one participant explained:

“My husband was a big card player. He had a bunch of guys [over] and this guy goes ‘Jeez, I’ve seen you before. Where have I seen you before?’ And I’m like, I don’t know. And all of a sudden it clicked that he was a counseling client. … And then I went upstairs and never went back downstairs after because I knew that he then clicked that we knew each other. So I knew I had to keep my distance … keep my space and let him not feel uncomfortable” (Participant 15).

Participants consistently emphasized the importance of patient confidentiality, but some believed in having more flexible boundaries for community engagement. They believed that actively participating in their communities allowed people to become better acquainted and effectively spread the word about their programs or services. As one participant stated:

“I actually encourage dual relationships, but properly managed. And that’s because in a rural community if you don’t attend some community events, if you aren’t involved, if you are keeping everybody at arm’s length, you’re considered standoffish, you’re considered aloof, you’re considered an outsider” (Participant 13).

Most providers believed that managing dual relationships was necessary. In their view, being integrated into the community helped to establish trust. Others mentioned that they would offer workshops and seminars as a way to reach out to residents and rely on advertisements in local newspapers in addition to social media. One participant explained, “So we have to realize that we maybe need to go out to the community a little bit more, rather than them having to come to us. We have to think about all the ways we could reach them” (Participant 1).

### 3.5. Community Trauma

Participants described how traumatic losses, such as suicide, the mass euthanasia of an entire herd of cattle, or an accident could profoundly affect the entire community. We labeled this theme “community trauma” to reflect the collective psychological impact experienced due to significant and distressing events. The close-knit nature of rural areas often meant that traumatic losses were shared experiences of distress and grief, disrupting the community’s social fabric, sense of safety, and trust in institutions. One significant issue highlighted by participants was the limited mental health resources available in rural areas, which often resulted in different patients discussing the same issue. As one participant described, “The amount of opioid deaths that have been going on … and the increase here in the last little while has been felt by the entire community” (Participant 4).

Community trauma could manifest in other ways, including the erosion of vital support systems. As one participant noted, “There’s a lot of people who have been hurt by their church community. So they’re pulling away from that, but church community was kind of all they had left” (Participant 13). Participants described how community trauma of any type could be a catalyst to bring people together, but the effects were often short-lived and lacked the structure to have a lasting impact. For example, several participants described offering seminars or workshops, yet these were often poorly attended and lacked continuity. Often the people who needed the support were not the ones who attended, or the event took place at an inconvenient time for farmers. Working to establish ongoing mental health support was essential. As one participant described,

“You did a one off and everybody’s like, great. But then, now what? You have nothing happening after and I think that becomes where you really get a disconnect because like you’re doing something and then you stop and then you do something and they stop and then you’ve like, lost all those people in between” (Participant 1).

### 3.6. Four-Quadrant Model

**Figure 1:**
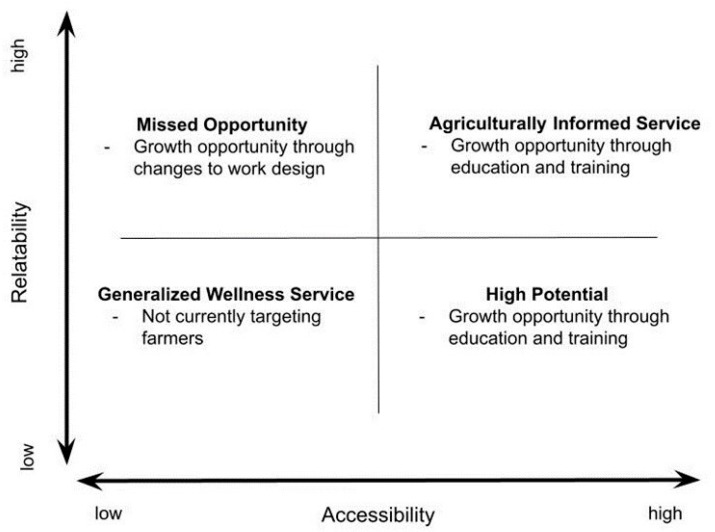
The four-quadrant Agriculturally Informed Service Model.

One of the final steps in our analysis involved identifying the fundamental structure of the themes we identified. Upon reviewing our coding, two themes emerged as essential considerations for healthcare providers delivering mental health services to farming populations in rural areas: Relatability and Accessibility. As illustrated in Figure 1, we represented these themes in a four-quadrant model, which served as a basic framework for identifying the placement of healthcare services concerning the provision of agriculturally informed rural mental healthcare. We labeled this model, the Agriculturally Informed Service Model.

Utilizing this framework, we categorized participants’ services into one of the four quadrants, assigning each a label and description. For instance, the Missed Opportunity quadrant (high relatability, low accessibility) encompassed services where mental healthcare providers understood farm culture and could deliver farm-informed care. However, structural barriers such as limited work hours or reliance on virtual care options hindered accessibility. The High Potential quadrant (low relatability, high accessibility) delineated services where mental healthcare providers offered flexible hours and policies. However, they lacked sufficient knowledge of farm culture to confidently provide agriculturally informed care. The agriculturally informed quadrant depicted services where providers possessed a strong understanding of farm culture. Additionally, their service structures were flexible, offering various options and accessible hours.

### 3.7. Evidence-Based Guiding Framework

**Figure 2:**
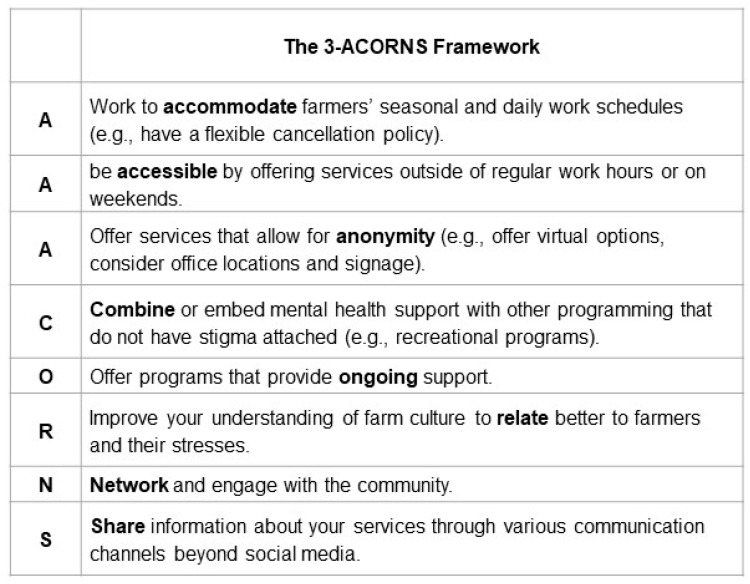
Evidence-based 3-ACORNS framework.

We identified eight practices associated with healthcare service providers who offered agriculturally informed services and successfully engaged with farmers (Figure 2). We named this framework the 3-ACORNS framework, representing the acronym AAACORNS. This framework extends our four-quadrant model by aiding mental healthcare providers aspiring to work with farmer clients in identifying aspects of their practice that may require adjustment. The metaphor of “acorns” resonated well in our Western Canadian context, drawing parallels with the bur oak, the only oak tree species thriving in the cold prairie climate. Similar to the bur oak’s resilience, we found that only agriculturally informed services demonstrated effective and long-term engagement with farming populations in rural areas.

## 4. Discussion

Healthcare providers acknowledged that farmers constitute a unique subculture, largely because of the occupational demands of the agricultural industry [19], within rural areas [37]. Consequently, participants in our study recognized the necessity for a distinct approach to mental health service delivery to address their needs. The current study aimed to understand the factors influencing mental help-seeking among rural-living farmers from the perspective of healthcare providers, while also exploring strategies they used to encourage help-seeking. Our findings offer insights for mental healthcare providers to adopt strategies aimed at improving help-seeking rates among farmers, thereby potentially reducing high levels of poor mental health and elevated suicide rates in this population [25,26]. Early engagement with appropriate support has been shown to mitigate distress and suicidality, a pressing concern in farming populations [42,43,44]. Our analysis revealed five major themes among mental healthcare providers: ensuring accessibility, being relatable, addressing stoicism and stigma, navigating dual roles, and understanding community trauma. These themes are interrelated, illustrating how an understanding of these issues can inform the adaptation of healthcare practices to provide more agriculturally informed services to facilitate farmers’ help-seeking.

A prominent theme emerging from our data highlighted the pressing need for accessible mental healthcare services in rural areas. Healthcare providers emphasized the challenges farmers face in accessing these services, citing lengthy travel times on rural and remote roads, a preference for in-person options over internet-based ones, and the financial strain of out-of-pocket expenses. Our findings align with Hagen et al. [35], who also emphasized the significant barriers posed by financial constraints and geographical distance to accessing mental health services in rural areas. While technology-based services hold promise in addressing accessibility barriers, unreliable internet connections in rural areas continue to hinder the establishment of telemedicine services [32,34]. For instance, in Alberta, approximately 67% of rural residents lack access to reliable high-speed internet [45]. Additionally, Vayro et al. [33] reported that general practitioners (GPs) were hesitant to refer patients to technology-based solutions due to concerns about their relevance and suitability for farmers. This point highlights the importance of tailoring such services to meet the specific needs and preferences of farming communities to improve help-seeking behavior. To address these challenges, healthcare providers in our study offered several service options, flexible cancelation policies, and extended service hours into evenings and weekends.

Relatability emerged as a significant concern for healthcare providers aiming to encourage farmers to utilize their services. However, ensuring that mental health resources are culturally relevant for farmers has been overlooked. One review found that 62.6% of programs aimed at increasing mental health resources for farmers focused on providing educational materials such as fact sheets, web pages, workshops, conferences, and articles [46]. As our participants noted, these types of resources are likely to have little or short-term impact because they lack interaction and continuity. While having someone to talk to was deemed important, the review also found that many hotlines or in-person counseling services made available for farmers tended to offer general services instead of ones tailored to their mental health needs [46]. The importance of ensuring mental health services is delivered by a provider with farm credibility is essential [34], particularly for individuals at an elevated risk for suicide and psychological distress. Our participants emphasized that being familiar with the community and possessing an understanding of agriculture and farm culture helped reduce the psychological distance that many of their patients may perceive when seeking professional help.

While opinions regarding the acceptability of dual relationships varied among participants, our findings underscored their importance in rural settings. Consistent with previous research [47,48], integrating into the community fostered trust and facilitated cultural competence development among healthcare providers [49]. Halverson and Brownlee [48] similarly argued for the necessity of dual relationships in rural areas due to resource limitations. However, participants acknowledged the challenge of maintaining such relationships, given the frequent encounters with patients in various community contexts, although they believed that clear boundaries could mitigate potential issues. Furthermore, the complexity of maintaining dual relationships in rural areas could be exacerbated during critical events such as suicides, which impacted multiple families. Farming communities, often regarded as collectivist societies within the broader individualistic culture [50], experienced collective grief and loss in such situations. Unlike their urban counterparts, our participants highlighted the need for careful consideration of confidentiality and privacy issues in rural settings.

Our study contributes to a growing body of research aimed at addressing help-seeking behaviors among farming populations in rural areas. A notable strength of our study is the four-quadrant model and guiding 3-ACORNS framework. To our knowledge, no such model exists to assist providers in assessing whether their practices align with agriculturally informed approaches. As such, we will translate our findings and share them with healthcare and social service organizations, counseling practices, and policymakers so that they have a better understanding of the needs of farmers and the strategies that could be adopted to make an organization or practice more agriculturally informed.

There are also implications for intervention development, clinical practice, and future research. First, given the persistent mental health stigma in many rural areas [51], healthcare providers may need to think creatively about how to incrementally deliver mental health services. For instance, while single workshops or seminars may not be effective in producing long-term change, they can serve as a starting point for spreading awareness and building trust within the community. Additionally, integrating mental health programming within existing services, such as recreational programs, may present an effective strategy to combat stigma while promoting awareness and engagement [51,52]. We do, however, recognize that implementing changes to one’s practice is highly dependent on the type of practice. For example, providers who operate a private practice have more flexibility to make changes, whereas providers working for a healthcare organization have less. Second, healthcare providers practicing in rural areas should receive training and education related to farmer mental health issues to enhance their cultural competence and ability to detect signs of poor mental health during consultations. Finally, our study focused on the job design factors and work approaches to engaging with farmer clients. While these factors are important to encourage initial help-seeking, more research is needed to understand how to foster trusting relationships in the therapeutic setting so that farmers return to continue their mental health work.

However, our study has limitations. Despite using a random sampling approach, there may be a self-selection bias. It is possible that participants who were aware of disparities in urban–rural healthcare services were more inclined to participate. Additionally, our sample included more women participants from a Western Canadian province, which may limit the generalizability of the findings. Further research is needed to validate our findings across diverse geographical and political contexts. It may also be beneficial for quantitative studies to explore the relative importance of service-related factors in predicting help-seeking behaviors among farmers and identify individual differences variables. Such investigations would further inform the development of tailored approaches to enhance farmers’ help-seeking behavior.

## 5. Conclusions

In summary, our study provides new insights for improving mental healthcare delivery for farming populations in rural areas. By identifying key strategies and approaches used by mental healthcare providers, our findings contribute to ongoing efforts to improve help-seeking among farmers, with the ultimate goal of reducing the high levels of poor mental health and suicide prevalent in this demographic. Our evidence-based framework, rooted in five major themes, offers a roadmap for adapting mental healthcare services to address the unique challenges faced by farmers. By addressing the issues of accessibility, relatability, stoicism, stigma, dual roles, and community trauma, our findings highlight the complexity of delivering mental healthcare in rural areas. Moving forward, understanding and integrating these insights into healthcare practice can significantly impact early engagement with appropriate support, thereby mitigating distress and suicidality among farming populations.

## Data Availability

The interview guide and de-identified data presented in this study are available upon request from the corresponding author. The data are not publicly available due to privacy concerns.
